# Correlates of Cooperation in a One-Shot High-Stakes Televised Prisoners' Dilemma

**DOI:** 10.1371/journal.pone.0033344

**Published:** 2012-04-02

**Authors:** Maxwell N. Burton-Chellew, Stuart A. West

**Affiliations:** Department of Zoology, The University of Oxford, Oxford, United Kingdom; German Primate Centre, Germany

## Abstract

Explaining cooperation between non-relatives is a puzzle for both evolutionary biology and the social sciences. In humans, cooperation is often studied in a laboratory setting using economic games such as the prisoners' dilemma. However, such experiments are sometimes criticized for being played for low stakes and by misrepresentative student samples. Golden balls is a televised game show that uses the prisoners' dilemma, with a diverse range of participants, often playing for very large stakes. We use this non-experimental dataset to investigate the factors that influence cooperation when “playing” for considerably larger stakes than found in economic experiments. The game show has earlier stages that allow for an analysis of lying and voting decisions. We found that contestants were sensitive to the stakes involved, cooperating less when the stakes were larger in both absolute and relative terms. We also found that older contestants were more likely to cooperate, that liars received less cooperative behavior, but only if they told a certain type of lie, and that physical contact was associated with reduced cooperation, whereas laughter and promises were reliable signals or cues of cooperation, but were not necessarily detected.

## Introduction

The evolution and maintenance of cooperation poses a problem for both Darwinian selection and economic models based on a rational actor (“*Homo economicus*”) that solely aims to maximize personal income [1]–[4]. The problem is that cooperative behaviors benefit other individuals, and so individuals that do not cooperate should be able to out-compete cooperators. Hamilton's influential theory of inclusive fitness explains how cooperation can be favoured between those that share genes for cooperation [3], [5], [6]. Yet cooperation also occurs between genetically unrelated individuals and even between individuals from different species [7]–[10].

The inherent instability of such cooperation between non-relatives is often conceptualised with the aid of the prisoners' dilemma or the tragedy of the commons whereby individuals do best by not cooperating, no matter what their opponents do [11], [12]. Put in game theory terms, the ‘Defect’ strategy is dominant, because it always leads to a higher payoff (or at least never a worse payoff) than employing ‘Cooperate’. This results in an inevitable outcome (hence ‘tragic’) in which all rational actors defect, even though collectively they would all be better off if they had all cooperated, hence the dilemma [11], [12].

In humans, cooperation is often studied in a laboratory setting using economic games, such as the prisoners' dilemma or some multi-player variant framed as a Public Goods game [13]–[17]. Such laboratory experiments are sometimes criticized for being played for low stakes (typically around 2 or 3 hours wages) by non-representative student samples and thus the conclusions drawn are accused of lacking external validity and being biased towards more pro-social outcomes [18]–[25].

**Figure 1:**
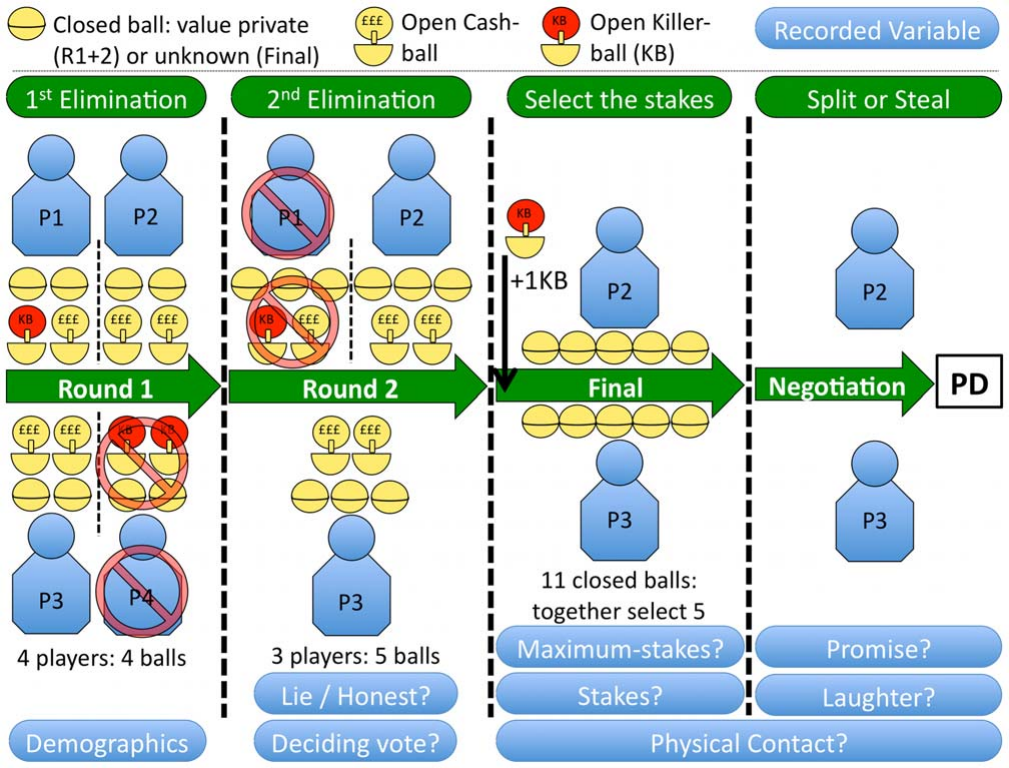
A summary of the game show procedure showing the distribution of balls and players by round, along with when certain variables were observed and recorded. The following text is a brief glossary followed by detailed description of each round. **Golden balls**: the ‘golden’ spheres that contain either cash values inside (‘£££’) or the word ‘killer’, in which case they are a ‘killer ball’. **- £££ Cash balls:** range in value from £10 to £75,000. **- Killer balls:** have no positive value and are very damaging because they each reduce the current prize fund by 90% if they end up in the final selection. **Stakes:** the final prize fund, derived from 5 randomly selected balls in the final round. **Maximum-stakes**: the maximum possible prize-fund available at the start of the final round, calculated from the 5 most valuable balls in the final round. **Round 1:** The game starts with 12 cash balls and 4 killer balls. Each contestant receives four balls at random, and must place two, at random, on the ‘Front Row’, which is public and keeps two on the ‘Back Row’, which is private. In this hypothetical example, Contestant 4 has received two killer balls on her front row and Contestant 1 has one killer ball, therefore one of the eight private balls must be a killer ball. The four contestants each show their public balls, and then take turns declaring their private balls, where they are free to lie. The contestants then each vote one contestant off from the game. The evicted contestant leaves the game, along with their four golden balls, which are ‘binned’. The best outcome for the group is to eliminate the contestant with the ‘worst’ balls. After the votes have been counted, each contestant must show the true value of their private balls, this way any liars are exposed. **Round 2:** The 12 balls remaining from Round 1 are carried through to Round 2, where they are mixed with an extra three balls, to provide a total of 15 golden balls that are randomly distributed once more. Once again the contestants declare the value of their private balls, and once again are free to lie, before voting eliminates another contestant and their balls. We only analysed lying behavior in this round. **Final:** The two surviving contestants and their 10 balls enter the final, where they are accompanied by one additional killer ball. The contestants are clearly told what the maximum prize fund possible is (Maximum-stakes) before selecting five of the 11 golden balls at random to determine their final prize-fund (Stakes). **Negotiation:** After the prize-fund has been determined, the two contestants are given some time (typically under 1 minute) to discuss and negotiate their decision in the simultaneous prisoners' dilemma. **PD - Split or Steal:** the contestants now play a mini-game, which resembles the prisoners' dilemma and is referred to by the show as “Split or Steal” (see Table 1 for payoff details).

Golden balls is a televised game show (see Figure 1 for a summary) that uses the prisoners' dilemma, with a diverse range of participants, often playing for very large stakes (often equivalent to more than a year's average salary). Specifically, the show ends with two contestants making a simultaneous decision to either split (cooperate) or steal (defect) a sum of money. If they both choose to split, they share the money equitably, but if only one contestant chooses steal then this contestant gets to take all the money. However if both contestants choose steal, they both receive £0. To steal therefore fits the requirements for a *weakly* dominant strategy [13], [26]. This is because it is always either the best (when opponent splits) or equal-best (when opponent steals) response to an opponent's decision, and is therefore the best strategy to employ in terms of maximizing income (see Table 1) [13], [26]. This version of the game is therefore known as the ‘weak’ prisoners' dilemma and to defect is still the best strategy to employ in a one-shot weak prisoners' dilemma just as it is normally [13], [26].

**Table 1:** The payoff matrix for the final decision in the game show.

	*Opponent decision*	
Focal Player Decision	*SPLIT*	*STEAL*
**SPLIT**(**payoff**/*opponent payoff*)	**HALF**/*HALF*	**ZERO**/*ALL*
**STEAL**(**payoff**/*opponent payoff*)	**ALL**/*ZERO*	**ZERO**/*ZERO*

The payoffs refer to ‘ALL’ the prize-fund, ‘HALF’ the prize-fund, or ‘ZERO’, i.e. £0.

The payoffs conform to a *weak* prisoner's dilemma, whereby to STEAL pays better or at least as well as to SPLIT, regardless of what one's opponent chooses.

The show also has two earlier rounds, whereby the contestants simultaneously vote to evict one contestant from the show per round, thus four contestants are whittled down to the two finalists who play the prisoners' dilemma. Contestants make their votes after being informed by a mixture of public and private information on the worth of each contestant's golden balls. Depending on the round, each contestant has received either four (round one) or five (round two) golden balls, with the worth of two balls on public display, and the others only known privately. The balls have been allocated at random from a population of balls ranging in value from a minimum of £10 to a maximum of £75,000. There are also some severely damaging special balls, termed “killer balls”, that are of no monetary value and that reduce the final prize fund by 90% if not eliminated. Each ball has potential implications for the final prize fund, thus the group collectively benefits by elucidating the true value of the balls and eliminating the worst balls from the game. They can do this by identifying which contestant has the worst balls and voting that contestant, along with his/her balls, off the show. The final prize fund is derived from the golden balls that are carried through to the final by the two surviving contestants. Before the voting takes place, contestants declare the value of their hidden balls (their private information). The contestants are free to lie, although they are forced to declare the truth at the end of each round, after voting has taken place. A rational, income maximizing, contestant should therefore aim for the following; (1) to secure his or her own passage to the final, (2) to maximize the final prize fund (by voting out contestants with low value golden balls and/or killer balls), and (3) to pair himself or herself with a cooperative contestant in the final round. Of course these multiple goals may not be mutually compatible. Such a structure of the earlier rounds allows us to analyse the frequency and consequences of lying versus honest behavior.

We use data from this television show to: (1) examine the extent to which individuals cooperate when ‘playing’ for large stakes; and (2) determine the correlates or cues of cooperation. Our aim is to provide observational data from a game with high stakes, to both complement existing experimental studies and suggest new issues that would benefit from experimental study, analogous to the use of observational field studies in the field of animal behavior [27]. Game theoretical predictions of how individuals will behave depend upon what individuals are trying to maximise (their ‘utility’ function), which can depend upon income, but also factors such as the welfare of others or an aversion to inequity [28]–[30]. However, our aim is to determine the factors of their environment, including their opponent's phenotype, which contestants are responding to, and not to test strong *a priori* predictions. We use game theory models based on a rational *Homo economicus* and evolutionary theory to guide this search, because they suggest correlates that could be important. Our starting point is that each contestants desires that his or her opponent splits, and thus should behave in such a way that he or she thinks will maximize the probability of such an outcome.

Since submitting our results to peer review we have learned of another study, by van den Assem *et. al.* that uses the same data set to address similar questions, and has now been accepted for publication in Management Science [31]. Their dataset is slightly larger than ours (five versus three series) because more episodes had been filmed when they started their study and thus their results are not expected to be exactly the same. In addition we take a more biological viewpoint and thus only we analyse the effects of geographical distance, laughter and physical contact between opponents (see Methods below). We also are unique in presenting the data from the pre-game interviews that reveals contestants' declared preferences and strategies (see Methods below).

## Methods

### Data Collection

We purchased all 150 episodes (series 1, 2, and 3, first aired March 2007–May 2008) that were available at the time in DVD format from Endemol UK in October 2008. One episode was a duplicate. We also excluded six episodes because they featured contestants that had been on before. Thus our samples size is 143 episodes, featuring the decisions and behavior of 286 contestants.

### Data scoring

Every episode was watched in real-time by one of us (MNB-C), who recorded variables without knowledge of the final outcome of the episode. The variables can be grouped, for the purposes of presentation (the analysis is not affected by their groupings), into three sets. These sets are: (i) Contestant demographics, which are variables that are fixed at the start of an episode for each contestant and their opponent; (ii) Structural variables, which develop as the episode proceeds but are not ‘behavioral’, these could be said to make up the environment contestants experience (excluding the phenotypes of their opponents); and (iii) Behavioral variables, which are the actions of the contestants and their opponents. A summary of the variables and when they were recorded can be seen in Figure 1.

### Contestant preferences

Our dataset allowed us to score the preferences of the contestants before they played the game and to compare these with their final decisions, thus we could compare the frequencies of different preferences, different choices, and consistency of preferences. We could do this because before the game begins the contestants are interviewed in private and asked to talk about how they will play the game. The contestants' responses are free-form and therefore not every contestant specifies what decision they will make in the final game (if they get that far) - we did not include this variable in the full model of the main analysis because it was not available for all contestants.

### Contestant demographics (6 variables)

We recorded the age and sex of each contestant (“Age” and “Sex” respectively) and for their opponent (“Opponent-Age” and “Opponent-Sex” respectively). We also used the given home location of each contestant and their opponent to calculate the distance between the two (“Distance”). Finally, we also noted which series their episode was from in order to control for any changes over time as later contestants are possibly influenced by viewing earlier series (“Series”). We did not use episode number as is used by van den Assem *et. al.* [31], for each series was recorded in full before been shown, therefore contestants from relatively later episodes within a series were not exposed to more episodes than contestants from relatively earlier episodes. Distance was calculated by taking the shortest automobile route calculated on GoogleMaps. We accept that such measurements are imperfect.

### Structural variables (3 variables)

The contestants play for a prize fund at the end of the game. This prize fund is determined by the picking of five golden balls at random from a selection of 11 (see Figure 1). The contestants and viewers are always and repeatedly made aware of the maximum potential prize fund possible, which is the sum of the five most valuable balls. To test if contestants are sensitive to both the prize fund and the prize fund as a proportion of what they could have won, we recorded both the prize fund contested (“Stakes”) and the maximal potential prize fund possible (“Maximum-stakes”). Prior to the final round of the game (see Figure 1), there are three contestants competing for the final two places. In this stage, the contestants each vote one contestant off, and typically two contestants will cancel each other's votes out by voting for each other, thus leaving the other contestant with the deciding vote. This dynamic is often ‘common knowledge’. We tested to see if these “*Deciders*” as we called them, reaped any benefits from their status in the form of increased rates of splitting by their opponent in the final, and if their status affected their own decision making.

### Behavioral variables (8 variables)

For each contestant we recorded if they, or their opponent, lied or not (“Lie”, “Opponent-lie” respectively) in the round preceding the final decision to split or steal. We also recorded if they, or their opponent, initiated any physical contact during the final round or negotiation stage (“Touch”, “Opponent-touch” respectively). We also recorded if, during the negotiation stage, which occurs immediately after the stakes have been determined and immediately prior to the final decision to split or steal, contestants or their opponents made a promise to split (“Promise”, “Opponent-Promise” respectively), and if they, or their opponent, initiated any laughter (“Laugh”, “Opponent-laugh” respectively).

Lies came in two types, contestants could lie by denying they had a killer-ball (and that it was a cash-ball instead), or they could inflate the values of their cash –balls (see Figure 1). Promises were only scored if a contestant explicitly stated that *they* would split. Specifically, the following definitive phrases were coded as promises; “*I will split*”, “*I am going to split*”, “*I promise to split*”. Phrases such as “*I want to split*”, “*we are going to split*”, “*we should split*”, and “*I came here to split*”, were not coded as promises. We accept that the term ‘Promise’ may not be the best fitting term for such a variable, and it could have maybe been termed ‘Commitment’ or some other term, but we believe this to be a semantic point only. Contestants that initiated laughter (during the final negotiation stage) were those that laughed but not in direct response to the laughter of their opponent. It was therefore possible for none, one, or both contestants to initiate some laughter. Touching was coded in a similar manner, as most physical contact was initiated by one contestant and completed/allowed by the other. Therefore, contestants that initiated physical contact were those that made the first or only move in a particular instance of contact. It was therefore possible for none, one, or both contestants to initiate an instance of physical contact.

### Analyses

We transformed the non-normally distributed variables ‘Age’, ‘Distance’, ‘Stakes’ and ‘Maximum-Stakes’ by taking their natural logarithms. As our response variable was binary (to ‘split’ or ‘steal’), we fitted binary logistic Generalized Linear Models (GLM), with errors clustered at the level of individual episodes, and tested for the significance of model effects using Wald's χ^2^ [32]. The final Minimum Adequate Model (MAM) was chosen based on a comparison of Akaike Information Criterion (AIC) scores [33]. Statistics reported are from the final model.

## Results

### What is the frequency of cooperation/defection?

Fifty percent of the 286 contestants chose “split” and 50 percent chose “steal”. If the choices of contestants were correlated or coordinated then one can predict the distribution of episodes resulting in a split-split, split-steal, or steal-steal outcome. This is done by using the observed frequency of split (P = 0.5) and steal (Q = 1−P) and by testing if the observed distribution differs significantly, using a chi-square test, from those predicted by p^2^+2pq+q^2^ = 1. We found that the distribution of episodes was as one would expect by chance given the observed probability of splitting or stealing (39 episodes of split∶split, 39 of steal∶steal, and 65 of split∶steal, χ^2^_2_ = 1.182, P = 0.554), suggesting that the responses of the contestants were neither correlated nor coordinated.

### Are there different ‘types’ of contestant?

Fifty-nine percent of contestants (N = 148 of 252 interviewed) clearly stated in their interview whether they intended to split or steal in the final round, and they were equally likely to say that they would split (N = 68) or steal (N = 80, Fisher's exact test: P = 0.282). Females were less likely to express a clear intention to either split or steal (66 of 131 females versus 82 of 121 males, Fisher's exact test: P = 0.003), because, even though both sexes were equally likely to say they would split (38 of 131 females versus 30 of 121 males, Fisher's exact test: P = 0.480), females were less likely to say they would steal (28 of 131 females versus 52 of 121 males, Fisher's exact test: P = 0.0003). Seventy percent of the contestants (104 of 148) were true to their stated intentions, (the statement is not seen by other contestants so there is no strategic reason to lie), which is significantly greater than the proportion expected by chance (Binomial sign test: 104 successes in 148 trials, P<0.0001). The probability of playing as they had intended was the same whether the contestants stated they would split or steal (21 of 68 splitters subsequently chose steal versus 23 of 80 stealers who subsequently chose split, Fisher's exact test: P = 0.857), and whether they were female or male (22 of 66 females versus 22 of 82 males, Fisher's exact test: P = 0.470). Thus the ratio of splitters to stealers would appear to be equal as estimated by either contestants' hypothetical preferences or actual choices, although both types are equally likely to change their mind, with a probability of approximately 0.3 (95% CI: 0.23–0.38).

### Correlates of cooperation

The main statistical model is summarised in table 2 and all the results below are detailed therein.

**Table 2:** Binary logistic generalized linear model on the probability of Split versus Steal (N = 286, with errors clustered to 143 episodes).

Model Effect (Term)	Wald χ^2^	P	Beta	Odds ratio	Wald χ^2^	P	Beta	Odds ratio
*Contestant* *demographics*	Final model				Excluded from final model			
Age (log)^1^	9.12	0.003	−1.51	0.220				
Opponent-Age (log)	/	/	/	/	0.01	0.980	−0.01	0.988
Sex	3.90	0.048	−7.09	0.293				
Opponent-sex	0.50	0.478	−1.50	0.713				
Distance (log)	1.40	0.237	−0.67	0.513				
Series	2.10	0.148	0.14	1.147				
Sex*Distance^2^	3.77	0.052	0.85	/				
Sex*Opponent_sex	6.97	0.008	4.06	/				
Sex*Series	0.68	0.411	0.74	/				
Opponent_sex*Series	1.81	0.178	0.57	/				
Sex*Oppo_sex*Series^3^	8.72	0.003	−2.09	/				
*Structural variables*								
Stakes (log)	5.98	0.014	−2.66	1.208				
Max-stakes (log)	8.72	0.003	−2.78	0.567				
Stakes*Max-stakes^4^	6.86	0.009	0.27	/				
Decider	/	/	/	/	0.01	0.922	−0.03	0.968
*Behavioural variables*								
Lie	/	/	/	/	1.86	0.173	−0.42	0.656
Opponent-lie	/	/	/	/	0.07	0.798	−0.08	0.927
Promise^5^	21.10	0.001	1.41	4.095				
Opponent-promise	3.53	0.060	0.58	1.780				
Laugh^6^	9.92	0.002	1.09	2.975				
Opponent-laugh	/	/	/	/	1.25	0.264	0.41	1.510
Touch	1.05	0.305	−0.30	0.743				
Opponent-touch	0.10	0.749	0.75	2.114				
Touch*Oppo-touch^7^	5.77	0.016	−1.28	/				
Sex*Touch^8^	4.33	0.038	1.20	/				

1 Older contestants were more likely to SPLIT.

2 Males were more likely to SPLIT with geographically distant opponents.

3 Over time, females became both more likely to SPLIT with females and less likely to SPLIT with males, males showed no change over time.

4 STEALING was more likely as stakes increased but SPLITTING was more likely as the loss in potential winnings was increased.

5 Promising was a reliable cue of increased probability of SPLITTING.

6 Initiating laughter was a reliable cue of increased probability of SPLITTING.

7 Touching was a reliable cue of STEALING, and being touched induced STEALING.

8 Touching may only be a reliable cue of STEALING for males.

#### Contestant demographics

**Figure 2:**
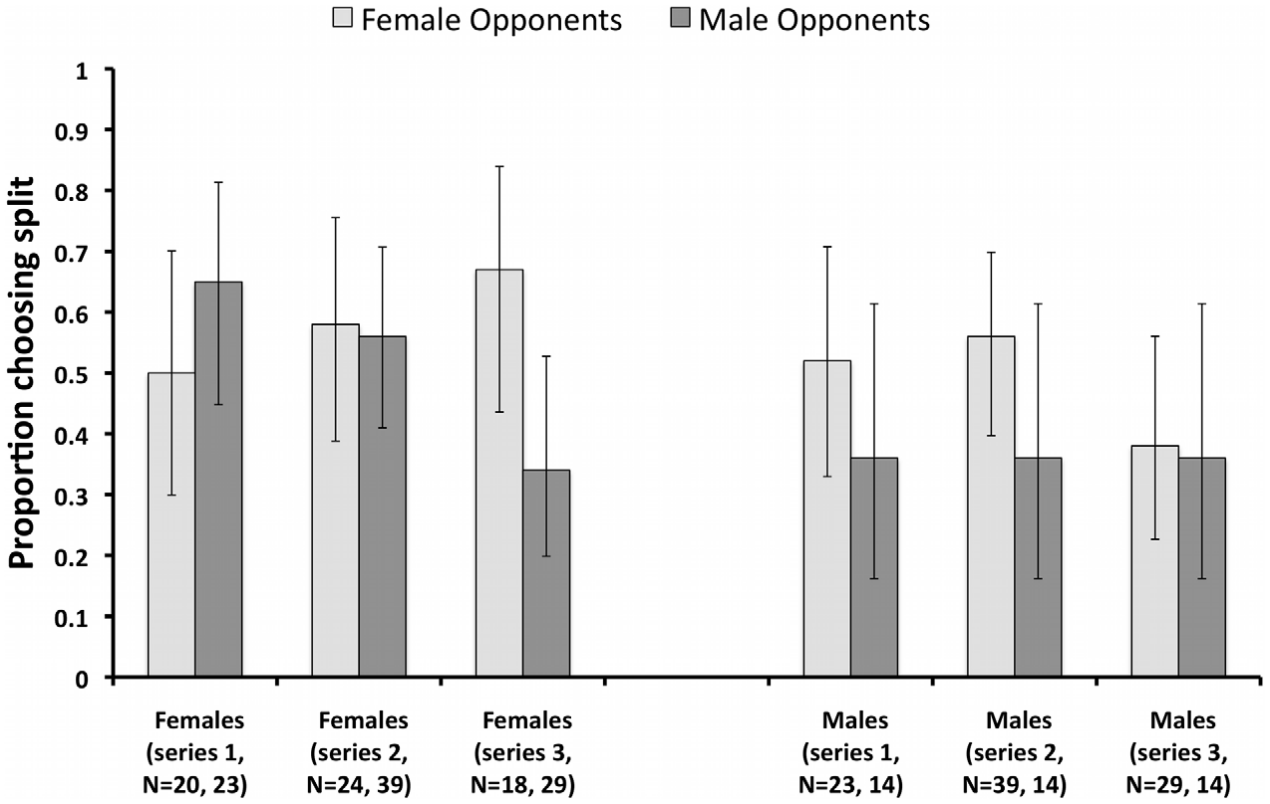
The proportion of splitting across series and depending on the sex of a contestant and the sex of their opponent. How contestants responded to their opponent's sex depended on their own sex and the series. Males (on the right) from different series behaved similarly but females (on the left) from later series were more cooperative to females and less cooperative to males compared to females from earlier seasons.

Older contestants were more likely to split (GLM: Wald χ^2^ = 9.12, P = 0.003), although contestants were not responsive to the their opponent's age (GLM: Wald χ^2^ = 0.01, P = 0.980). Overall, the sexes were equally likely to split when paired with the opposite sex (out of 91 pairings, females split 47 times and males 45 times) and appeared to prefer splitting with females (all female pairings were more cooperative than all male pairings). However, there was an interaction between the sexes that depended upon the season, with females becoming increasingly likely to split with females, and steal from males, in the later seasons (Wald χ^2^ = 8.72, P = 0.003, Figure 2). The distance between the two contestants current home-towns had no significant effect although there was a suggestion that male contestants were less cooperative with geographically close opponents (Wald χ^2^ = 3.77, P = 0.052).

#### Structural variables

**Figure 3:**
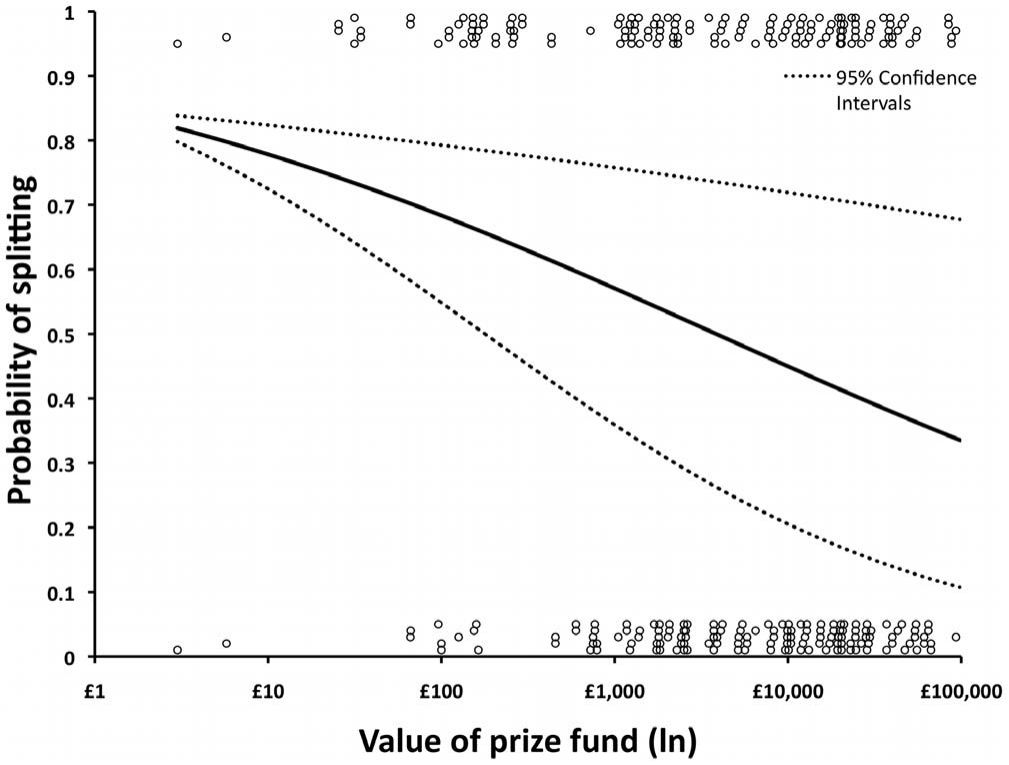
Logistic regression plot of the probability of a contestant splitting depending upon the value of the prize fund (Stakes). The probability of a contestant splitting decreased with larger stakes. The equation for the fitted response to the prize fund held the Maximal possible prize fund constant at the mean value of £40,324. The data have been separated, to improve visualization, by a process of shifting slightly from their true positions to slightly above 0 or slightly below 1.

The mean ± S.E.M and median Stakes were £14,094±£1,121, and £5,460 respectively, ranging from a minimum of £3 to a maximum of £93,250. The mean ± S.E.M and median Maximum-stakes available were £47,342±£1,676, and £40,000 respectively, ranging from a minimum of £5,000 to a maximum of £168,100. Contestants were sensitive to the size of the prize fund available, becoming more likely to steal when the stakes were larger (GLM: Wald χ^2^ = 5.98, P = 0.014, Figure 3), but there was an interaction between the stakes and the maximum-stakes possible, suggesting that for a given prize fund, contestants are more likely to split if the potential prize fund was larger (GLM: Wald χ^2^ = 6.86, P = 0.009). Deciders were no more or less likely to split, nor were the receivers of their deciding vote (GLM: Wald χ^2^ = 0.01, P = 0.922).

#### Behavioral variables

Contestants that either told a lie or were honest in the penultimate round were no more or less likely to split (GLM: Wald χ^2^ = 1.86, P = 0.173). Overall, contestants were also unaffected if their opponent had lied or not (GLM: Wald χ^2^ = 0.07, P = 0.798), but a re-run of the analysis with those that only told a lie to deny having a killer-ball excluded (N = 81), showed that those that inflated the value of their cash balls (N = 30) received less cooperation than honest contestants (GLM: Wald χ^2^ = 4.36, P = 0.037).

**Figure 4:**
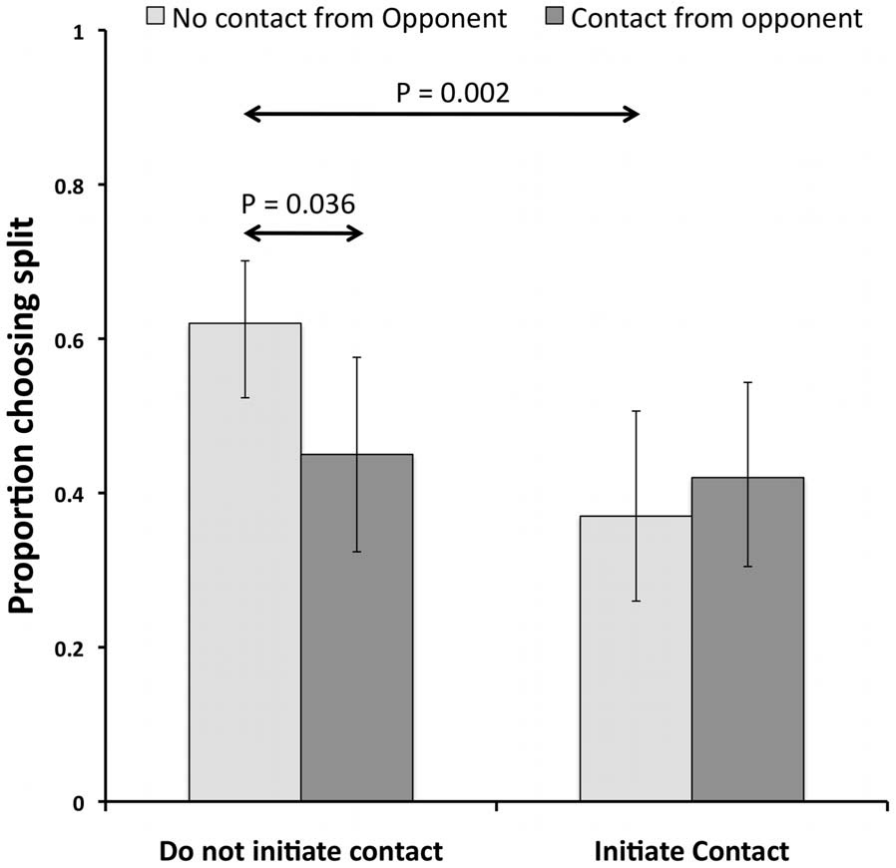
The proportion of splitting depending on whether a contestant initiated physical contact and/or whether their opponent initiated contact during the final two stages of the show. Contestants that were not involved in any physical contact were more likely to split than contestants that either initiated contact or were merely the receivers of such contact.

Contestants that ‘promised’ to split were more likely to do so (GLM: Wald χ^2^ = 21.10, P<0.001), but their promises only had a marginally non-significant effect upon increasing the likelihood that their opponents split (GLM: Wald χ^2^ = 3.53, P = 0.060). Contestants that initiated laughter were more likely to split (GLM: Wald χ^2^ = 9.92, P = 0.002), but as with promises, such laughter had no significant effect upon their opponents (GLM: Wald χ^2^ = 1.25, P = 0.264). There was a significant interaction between contestants that initiated physical contact or were simply the receivers of such physical contact, with contestants that neither initiated physical contact nor were touched, being more likely to split than anyone else (GLM: Wald χ^2^ = 5.77, P = 0.016, see Figure 4). Thus touching appeared to be a reliable cue of stealing, although perhaps only for Males (GLM: Wald χ^2^ = 4.33, P = 0.038).

## Discussion

We used data from the television game show Golden balls as an observational (non-experimental), high-stakes, prisoners' dilemma game. Contestants: (1) were sensitive to the size of the stakes, being more likely to steal if the prize-fund was relatively larger (Figure 3); (2) were more likely to steal if their opponent had inflated the value of their cash balls; (3) were more likely to split if they had initiated laughter, and more likely to steal if they had either touched their opponent or been touched by their opponent. The sexes were equally likely to split when playing against each other, but females appear to be shifting towards preferring to split with females and to steal with males (Figure 2).

### Why do the stakes matter?

Contestants were sensitive to the size of the stakes, and appeared to judge the value of the stakes in both absolute terms, and as a proportion of the maximal prize fund that was possible. This suggests that people use relative size heurestics to determine the true worth of money, an effect reviewed and termed “anchoring” by Ariely [34]. This result may have implications for the external validity of economic experiments played for typically small stakes, although of course it could be argued that our televised setting is just as problematic for external validity. Currently there is mixed evidence on the impact of raising the stakes for economic games, with many studies showing no effect, especially in the ultimatum game (where an effect is not necessarily expected because there are many equilibria), but an absence of proof is not proof, and it may just be that a sufficiently large change in stakes is required [25], [35]–[38].

### Contestant demographics and subject pools

We found that older contestants were more cooperative, and that the sexes appeared to treat each other differently. This has two implications for laboratory studies, whereby participants are typically university students and thus typically under 30 years old, and usually unaware of the identity and characteristics of their ‘opponents’. The first implication is that absolute levels of experimental cooperation may be under-estimates, but this should not be a problem as well designed studies aim to compare the response of participants to different treatments, rather than the absolute level of cooperation in particular treatments, and our data has nothing to say with regards to how different participants respond to different treatments [39]. The second implication is that the results of experiments may differ when participants are free to form opinions, perhaps stereotypical ones, about their opponents.

Just as laboratory experiments are criticized for only analyzing young students, this study can be criticized for studying a potentially non-random sample of the population [21], [22], [40], [41]. Although the contestants appeared to vary considerably on the show, they were a mixture of self-selected and commercially selected people. It would not be unreasonable to suggest that the personality types that are prepared to apply for the show are biased towards traits such as extroversion or sensation-seeking for example. Is this a problem? Boone et al's 1999 study examined the impact of personality on behavior in the prisoners' dilemma and found that “*personality matters*”, with ‘sensation-seekers’ and ‘self-monitors’ and those with an ‘internal locus of control’ showing more cooperation [42]. However, this result was driven by differences in the repeated version of the game, and there were no significant differences in the one-shot game, but one could also argue that our data do not come from a one-shot game due to the preceding rounds. Such an interaction was also shown for ‘self-monitors’ by Danheiser and Graziano's 1982 study [43]. Additionally, Rapoport's 1988 study found no difference, in a one-shot game, between “Professionals”, “Students”, and “Employees”, but found that “Business people” were marginally less cooperative, although of course professions are a crude proxy for personality [26].

Therefore the effect of personality on a one-shot encounter appears mixed at best, and a comparison between the overall result of our study (50% cooperation) matches well with the overall result of Rapoport's 1988 study, which found that 53% of 138 participants cooperated in a one-shot weak prisoners' dilemma [26]. Furthermore, our result is consistent with that found in the first round of play of many economic experiments, and the overall mean of 47% calculated from 130 experiments reported in Sally's 1995 [16] meta analysis of the prisoners' dilemma and the 51% reported by Ledyard's 1995 survey [44]. Thus our subject pool would appear to by reasonably ‘typical’ for such a perhaps ‘WEIRD’ (Western Educated, Industrialised, Rich, and Democratic) sample [40], [41].

### Honesty and honest signals of cooperation

Contestants could presumably increase their chances of making the final round by lying about their private information, but if they lied by inflating the value of their cash-balls they suffered from reduced cooperation, perhaps as a result of anger or reduced trust from their opponent. This may explain why only 82 of the 429 contestants in the penultimate round chose to lie about their cash balls. In contrast, contestants that lied by denying having killer-balls were not treated differently, perhaps because their lies were seen as ‘understandable’, as the only viable course of action open to a contestant with a ‘bad hand’.

The correlation between initiating laughter and cooperating is consistent with the idea that laughter and smiling function as honest signals of cooperation [45]–[50]. Such honest signals can be explained if they are too costly for defectors (by the ‘handicap principle’) [51]–[53] or if they are linked genetically by the same, or tightly linked, gene(s) (the ‘green beard’ mechanism) [54], [55]. However laughter is presumably very cheap, for both cooperators and defectors, and there is no good reason to believe or expect that laughter and cooperating are linked genetically [54]. Alternatively, laughter may generally serve to signal a desire to enter into a mutualistic arrangement or to signal appeasement [56], a situation whereby the signaler and receiver both favour the same outcome [57]. Such a signal would not be adaptive within a one-shot prisoners' dilemma, because the lack of repeated interactions would remove the shared interest between interactants. It is therefore difficult to discern if our contestants were able to read such signals as suggested by results of other studies [58], [59]; but the significant role of non-verbal communication in ensuring trust and cooperation has been shown elsewhere [50].

The same argument applies to the function and value of promises. Even though non-binding promises in a one-shot interaction should be worthless we found that those contestants that explicitly stated or promised that they would split were more likely to split [13]. A similar result was reported in a Dutch sample of a similar show [18] and pre-play communication was shown to increase cooperation in the 1995 meta analysis by Sally [16]. However, once again we found that this potential signal of cooperation had no significant effect upon contestant's opponents. Which may explain why stealers did not merely lie and pretend that they were going to split.

Physical contact has been suggested to facilitate bonding through the release of endorphins [60], but it had an adverse effect in our data (see Figure 4), perhaps because whilst laughter is perhaps somewhat difficult to fake convincingly, reaching out and touching somebody is perhaps less so. In fact our contestants seemed to believe too much in the power of touch, and stealers were arguably prone to reach out in an attempt at manipulation, an attempt that perhaps merely served to signal insincerity.

How do the results of our study compare with those of van den Assem *et. al.* [31]? Both studies found that cooperation decreased as the stakes increased and that there was an “anchoring” effect of the maximal possible prize-fund, although van den Assem *et. al.* suggest that this effect no longer occurs in later series (series we do not have the data for). In addition, both studies found that “promises” were a reliable cue of cooperation but that they had no detectable effect on opponents. Both studies also found lying to be non-significant although we found that it depended on the type of lie, and we also found the above-described effects of laughter and physical contact. In contrast, van den Assem et al. found that contestants were less likely to cooperate with those that had tried to vote them off earlier in the show, whereas we analysed the voting behavior in a different way. In the penultimate round, with two contestants votes often cancelling each other out, we recorded who had the deciding vote and found that these individuals were no more or less likely to cooperate nor more or less likely receive cooperation. As contestants cannot positively vote ‘for’ another, but can only vote ‘against’ others, we suggest that this is why we found no positive effect, whereas van den Assem *et. al.* recorded votes against the focal player. They also recorded the voting behavior from round one (which we excluded to reduce complexity and uncertainty), and thus had a larger sample of negative votes. It is unclear whether this increased statistical power or a cognitive/behavioural bais in responding to negative votes over positive votes is responsible for the difference in results between the studies.

### To split or steal: a repeated game or a one-shot game?

We described the final game in our show, and the unit of our analysis, as a one-shot weak prisoners' dilemma, but is it really a one-shot game? Our result of 50% cooperation matches well with the 53% cooperation found in Rapoport's study on the one-shot weak prisoners' dilemma [26]. However perhaps our game should be seen as the end round of a repeated, multi-faceted game? How does our data compare to the final rounds in a finitely repeated prisoners' dilemma? Miller and Andreoni compared finitely repeated games (played with one partner for 10 rounds) and one-shot games (played one time with 10 sequential partners) and found that cooperation was higher in the finitely repeated game, in contrast to models based on rational backwards induction [15]. Although cooperation was higher, it declined rapidly towards the final rounds of the finite game, i.e. as the reputational benefits diminished. Cooper *et. al.* also compared a series of 10 one shot games versus a round 10 games with the same partner and found a similar result [61]. Both studies found that initial rates of cooperation were higher in the repeated games, at around 50–60% compared to 20–30%, but declined steeply in the final rounds of the repeated game to around 20%. It is interesting to note that a series of one-shot games with different partners appears to initiate lower levels of cooperation than in the single one-shot games of Rapport, suggesting a framing affect [26]. In comparison, our data would appear to more resemble the opening rounds of a repeated game rather than the final rounds, perhaps because our game is part of the repeated game of life, and the large television audience means that the wider reputational concerns do not diminish.

Of course if off-screen reputation concerns are so important then this begs the question of why cooperation is not even higher? One reason is that reputations can also be enhanced by punishing and perhaps merely by not cooperating with cheats in certain circumstances [62]. Or perhaps some participants are simply less concerned about their off-screen reputation. Alternatively, they may feel that the game-show setting justifies a more competitive approach and that they can plausibly argue to their peers that their in-game behavior is no guide to their real-world social behavior. It would be interesting if laboratory experiments could explicitly test the effect of framing experiments involving social dilemmas as contests versus moral decisions. Intriguingly it has already been shown that merely naming the prisoners' dilemma as the Wall Street game reduces cooperation to around 33% compared to around 66% when named the Community game [63]. Of course part of the commercial success of Golden balls is due to its deliberate mixing and blurring of frames in order to invoke competing behavioural norms (‘normative conflict’) [64] and thus create more suspense for the audience and more discord for the participants.
